# Evaluating the Predictive Value of Post-Treatment Superb Microvascular Imaging for Complete Response to Neoadjuvant Chemotherapy in Invasive Breast Cancer

**DOI:** 10.3390/bioengineering13040449

**Published:** 2026-04-11

**Authors:** Rana Gunoz Comert, Ravza Yilmaz, Eda Cingoz, Zuhal Bayramoglu, Aysel Bayram, Baran Mollavelioglu, Mahmut Muslumanoglu, Ulas Bagci

**Affiliations:** 1 Department of Radiology, Istanbul Faculty of Medicine, Istanbul University, Istanbul 34093, Turkey; ravzaylmz@gmail.com (R.Y.); zuhal.bayramoglu@istanbul.edu.tr (Z.B.); 2 Radiology Department, Bagcilar Training and Research Hospital, Istanbul 34200, Turkey; edacanipek@gmail.com; 3 Department of Pathology, Istanbul Faculty of Medicine, Istanbul University, Istanbul 34093, Turkey; aysel.bayram@istanbul.edu.tr; 4 Department of General Surgery, Istanbul Faculty of Medicine, Istanbul University, Istanbul 34093, Turkey; baran.mollavelioglu@istanbul.edu.tr (B.M.); mahmutm@istanbul.edu.tr (M.M.); 5 Machine & Hybrid Intelligence Lab, Department of Radiology, Northwestern University, Chicago, IL 60611, USA; ulas.bagci@northwestern.edu

**Keywords:** breast cancer, superb microvascular imaging, magnetic resonance imaging, neoadjuvant chemotherapy, pathological complete response

## Abstract

**Purpose**: To compare the efficacy of Superb Microvascular Imaging (SMI) with grayscale ultrasound (US) and dynamic contrast-enhanced MRI in predicting pathological complete response (pCR) after neoadjuvant chemotherapy (NAC) in invasive breast cancer. **Methods**: A total of 115 patients included in the study were evaluated based on their pre-treatment imaging findings (US, mammography, and MRI). Following completion of NAC, all patients underwent grayscale US and SMI examinations. In patients with available post-NAC MRI, treatment response was additionally assessed by comparing MRI findings. Imaging results were correlated with postoperative pathological outcomes, which served as the reference standard. pCR was defined as the absence of residual invasive carcinoma, regardless of ductal carcinoma in situ. Molecular subtype, Ki-67, and axillary status were recorded. Statistical analyses included chi-square tests and stepwise multiple logistic regression. Significance was set at *p* < 0.05 (95% CI). **Results**: The median age was 51 years (range: 30–75). Most tumors were high-grade (55%) and invasive ductal carcinoma (95%). Breast-pCR was achieved in 43% of patients. Significant predictors of pCR included hormone receptor negativity, HER-2 positivity, high Ki-67 expression (≥40%), non-luminal subtype, and complete radiologic response on US and MRI (*p* < 0.05). Lower SMI index values were strongly associated with pCR (*p* < 0.001), with an optimal cut-off of 1.8 demonstrating good diagnostic performance (AUC = 0.804, 95% CI: 0.721–0.887). In multivariate analysis, the combined model including US, SMI, HER-2 status, and MRI showed the highest predictive performance (AUC = 0.890, 95% CI: 0.829–0.950), explaining 55.1% of the variance in pCR. **Conclusions**: An SMI index < 1.8, HER-2 positivity, and complete response on US and MRI are independent predictors of pCR after NAC. Combining SMI with multimodal imaging significantly improves predictive accuracy.

## 1. Introduction

Neoadjuvant chemotherapy (NAC), utilized for locally advanced or inoperable breast cancers to downstage the disease and facilitate more conservative breast and axillary surgeries, is also employed in the early stages of triple-negative and HER-2 positive aggressive breast cancers, where high pathological complete response (pCR) rates are anticipated based on current treatment protocols [1]. Pathological complete response (pCR) is a significant independent predictor of event-free survival and overall survival; thus, the ability to accurately predict pCR is crucial for optimizing clinical management and surgical planning [2,3]. Multiparametric breast ultrasound, encompassing elastography and Doppler examinations, is utilized for treatment monitoring to evaluate the response to NAC [4,5,6,7,8]. As macrovascular structures that supply nutrients to the tumor develop through neovascularization, they can be visualized using Doppler ultrasound. This imaging technique is employed to assess treatment response and to detect the presence of residual tumor [5,7]. However, Doppler examination has limited capability in visualizing slow-flow microvascular blood flow within the capillary bed. The Superb Microvascular Imaging (SMI) technique is distinguished by its ability to provide noninvasive, reproducible, and quantitative assessment of the capillary microvascular bed without the need for contrast agent administration, unlike CEUS [4,5,8,9,10]. During SMI examination, quantitative SMI index values, which represent the pixel-based Doppler signal ratio within the specified area, are obtained by tracing lesion boundaries or through the placement of regions of interest (ROI) [4,9].

Increased vascularity observed in SMI examination enhances grayscale ultrasound’s ability to differentiate between malignant and benign lesions [11,12,13,14,15,16]. High SMI index values have been associated with axillary lymph node metastasis and immunohistochemical markers related to vascularization, such as VEGF, ERG, and CD34, in the literature [17].

Numerous studies in the literature have explored predicting NAC treatment response using SMI, elastography, color/power Doppler, and contrast-enhanced ultrasound techniques performed either before or during the administration of NAC [4,5,6,7,8,18]. These studies primarily aimed to predict pathological response based on pre-treatment or early-treatment imaging findings. However, there is a notable absence of literature investigating the association between post-NAC SMI findings and pathological treatment response. In contrast to previous research, the present study specifically aimed to evaluate whether preoperative imaging findings obtained after completion of NAC could predict pCR.

## 2. Material and Methods

### 2.1. Study Design and Patient Cohort

In this retrospective single-center study, 162 female patients were included from a cohort of 534 patients diagnosed with breast cancer by core needle biopsy between January 2024 and December 2025, all of whom were selected for standard neoadjuvant chemotherapy by a multidisciplinary team.

**Figure 1:**
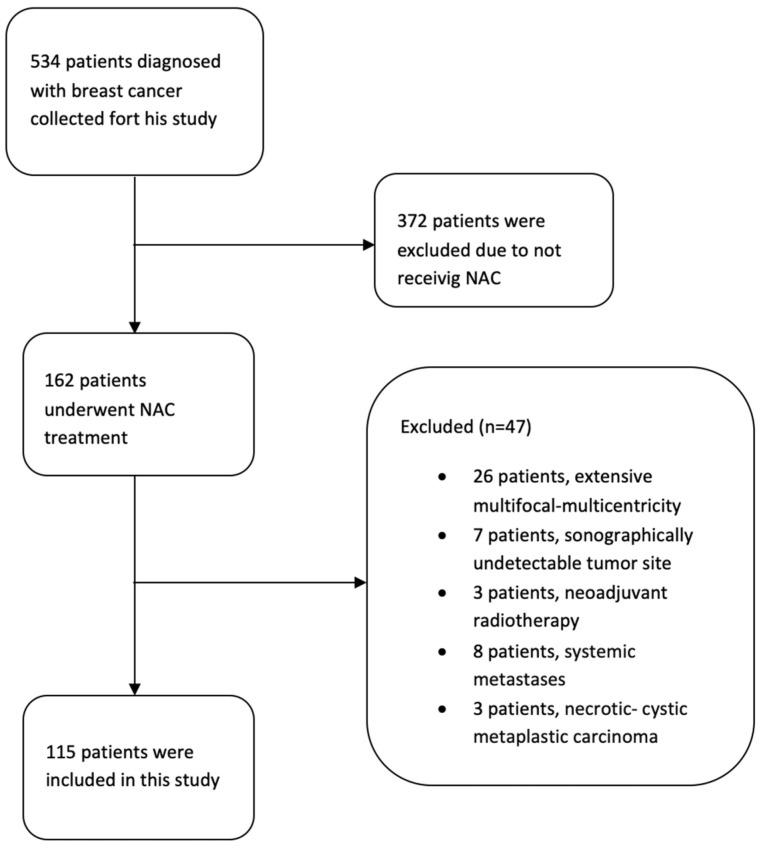
Flowchart of patient selection.

Twenty-six patients who were observed to have extensive multifocal, multicentric invasive foci; seven patients who exhibited a complete response to NAC and whose tumor sites could not be identified sonographically; three patients who received neoadjuvant radiotherapy; eight patients who could not undergo surgery due to systemic metastases detected during NAC; and three patients diagnosed with necrotic–cystic metaplastic carcinoma were excluded from the study *(n* = 47). Consequently, the examination results of *n* = 115 patients were analyzed (Figure 1).

### 2.2. Study Outcomes

The primary outcome was the ability of the SMI index to predict pathological complete response (pCR) after neoadjuvant chemotherapy, using postoperative histopathological findings as the reference standard.

Secondary outcomes included:

- Comparison of the diagnostic performance of SMI, grayscale ultrasound, and MRI in predicting pCR;
- Evaluation of the incremental value of combining imaging modalities (US, SMI, MRI) with clinicopathological parameters (including HER-2 status);
- Assessment of the association between the SMI index and tumor biological features, including molecular subtype, Ki-67 proliferation index, and axillary status.

### 2.3. NAC Regimens

According to the recommendations of the multidisciplinary oncology council, patients with HER-2 negative breast cancer (HR-positive or TNBC) received NAC consisting of doxorubicin and cyclophosphamide followed by paclitaxel. Patients with HER-2 positive disease were treated with docetaxel and carboplatin combined with trastuzumab, with pertuzumab added selectively based on individual clinical considerations.

### 2.4. Ultrasound and SMI Examination Technique

**Figure 2:**
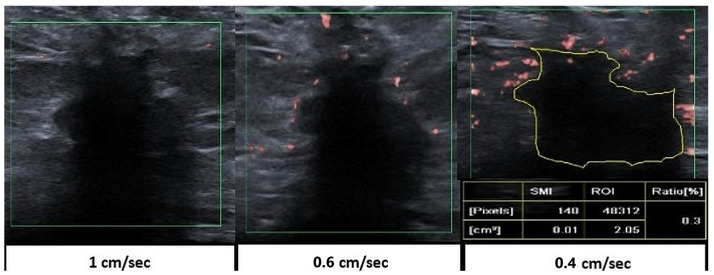
Superb Microvascular Imaging (SMI) assessment of the post-neoadjuvant chemotherapy (NAC) hypoechoic tumor bed. Perilesional flash artifacts are demonstrated at reduced velocity scale settings to enable detection of very-low-velocity flow signals within the hypoechoic tumor bed. During bedside ultrasound response evaluation, the SMI index value was obtained using a free-hand region-of-interest (ROI) technique, in which the borders of the hypoechoic tumor bed were manually delineated to achieve optimal boundary definition. In this study, measurements were taken within the velocity scale range of 0.4–0.6.

All patients underwent examination using the same ultrasound device (Aplio i800, Canon Medical Systems Corporation, Tokyo, Japan) equipped with a multi-frequency ultra widebandlinear (i18LX5) probe spanning the range of 7–18 MHz. All post-NAC SMI measurements were performed prospectively during patient care by two radiologists using identical technical parameters, without knowledge of the pathological response. Vascularity index measurements were obtained by manually delineating the hypoechoic tumor bed using a free-hand region-of-interest (ROI) method, excluding the surrounding tissue. The SMI index was automatically calculated by the ultrasound system as the ratio of Doppler signal pixels to the total number of pixels within the defined ROI, providing a size-independent measure of tumor microvascularity. In each patient, three SMI index measurements were performed and averaged. Two radiologists evaluated the images and reached a consensus for the final assessment. Cases with complete response in which the post-NAC tumor bed could not be clearly delineated were excluded from the study. Subsequently, post-NAC SMI index measurements, post-NAC grayscale ultrasound findings, post-NAC MRI response assessments, and postoperative pathological results were retrospectively reviewed through the PACS system. Following grayscale ultrasound evaluation after NAC, SMI was incorporated into the imaging protocol, and flow measurements were performed accordingly. Treatment response was assessed with reference to the patients’ mammography and MRI findings, as well as the histopathological results obtained from pre-NAC core biopsy specimens. Patients’ response to treatment on MRI and grayscale ultrasound was compared with pre-treatment imaging and categorized as complete/near complete, partial and no response. In the SMI examination, measurements were obtained within the dynamic range of 40–65, a frame rate per second of 36–60, and the color velocity scale ranging from 0.4 to 0.6 cm/sec permitting moderate flash artifacts in the perilesional area, provided that no intralesional flash artifact was present (Figure 2).

SMI examination was incorporated into the imaging protocol, with assessments performed by a consensus of two breast radiologists with 5 and 15 years of experience. Both radiologists evaluated pre- and post-treatment response with access to the patients’ ultrasound, mammography and MRI findings. All preoperative imaging assessments were performed without knowledge of the postoperative pathological outcomes. Treatment response on MRI and grayscale ultrasound was assessed by comparing post-treatment imaging to baseline studies and classified into three categories: complete/near-complete response, partial response, or no response.

**Figure 3:**
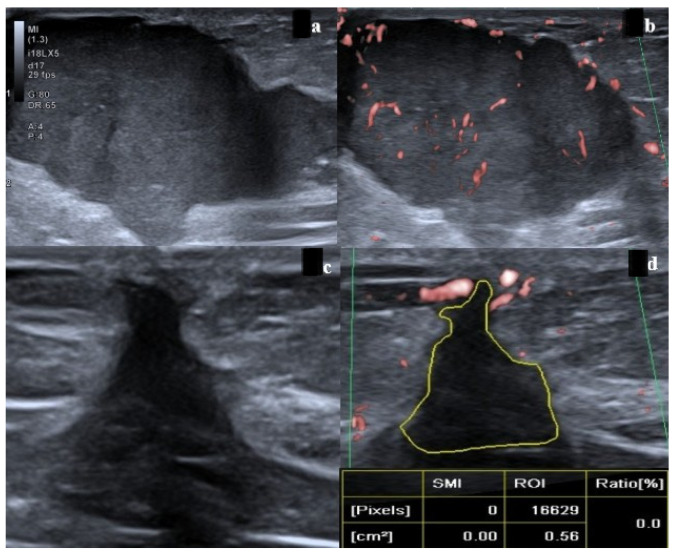

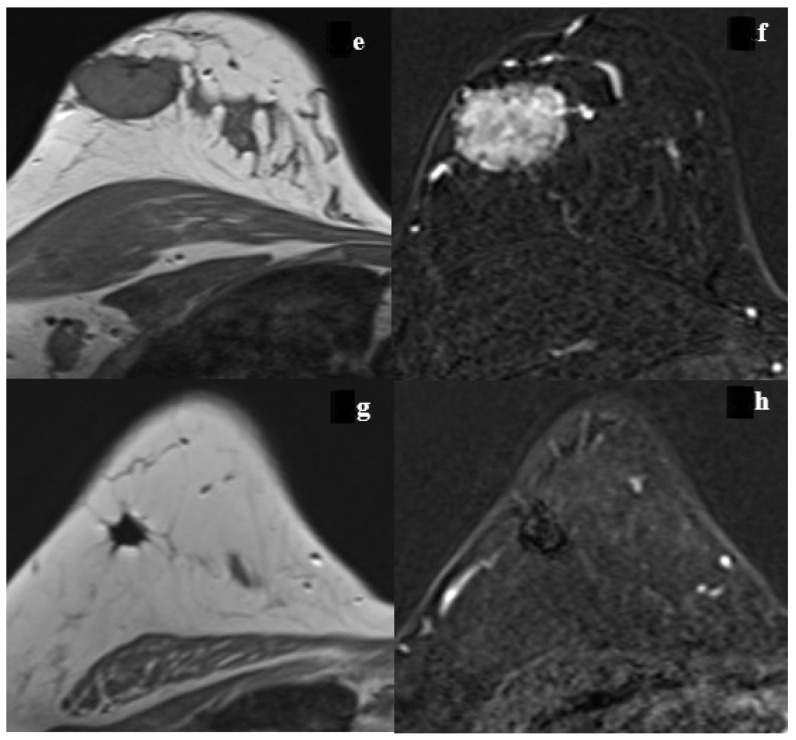
A 45-year-old female patient with a malignant mass located in the upper outer quadrant of the right breast, histopathologically confirmed as Estrogen Receptor (ER)-positive, Progesterone Receptor (PR)-positive, Human Epidermal Growth Receptor 2 (HER-2)-negative, Ki-67 40%, nuclear-grade-3 luminal B subtype. (**a**,**b**) Pre-neoadjuvant chemotherapy (NAC) grayscale ultrasound and pre-NAC Superb Microvascular Imaging (SMI) images. (**c**,**d**) Post-NAC grayscale ultrasound and post-NAC SMI images. On grayscale ultrasonography, although the lesion demonstrated marked concentric shrinkage following NAC, a residual mass configuration persisted and was therefore interpreted as a partial response. In contrast, SMI demonstrated no internal vascular signal within the hypoechoic tumor bed (SMI index: 0). Pre-NAC Magnetic Resonance Imaging (MRI) examinations demonstrate the mass on (**e**) T1-weighted (T1W) and (**f**) contrast-enhanced T1W subtraction images. Post-NAC MRI examinations show the tumor bed on (**g**) T1W and (**h**) contrast-enhanced T1W subtraction images. Following treatment, the tumor exhibited marked concentric shrinkage, and the absence of enhancement on dynamic subtraction images was interpreted as consistent with a complete radiologic response on MRI. Subsequent postoperative pathological evaluation revealed no residual invasive tumor or in situ carcinoma, confirming a pathological complete response (pCR).

**Figure 4:**
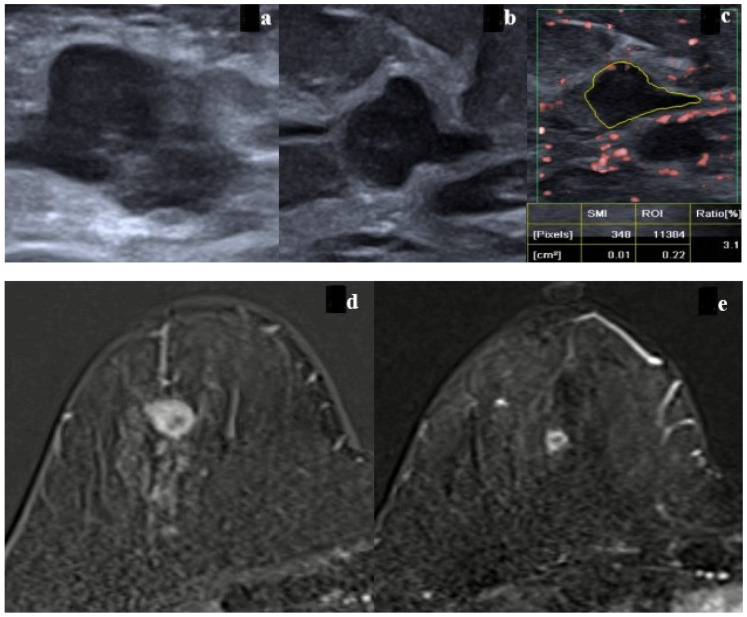
A 44-year-old female patient with an Estrogen Receptor (ER)-negative, Progesterone Receptor (PR)-negative, Human Epidermal Growth Receptor 2 (HER-2) 3+-positive, Ki-67 60%, nuclear-grade-3 malignant breast mass. (**a**) Pre-neoadjuvant chemotherapy (NAC) ultrasonography shows the mass. (**b**) Post-NAC ultrasonography (US) demonstrates concentric shrinkage; however, persistent convex mass morphology was interpreted as partial response. (**c**) Post-NAC Superb Microvascular Imaging (SMI) reveals internal vascularity within the tumor bed (SMI index: 3.1). (**d**) Pre-NAC contrast-enhanced T1-weighted MRI shows a heterogeneously enhancing mass. (**e**) Post-NAC Magnetic Resonance Imaging (MRI) demonstrates concentric shrinkage with persistent peripheral enhancement. Although US and MRI suggested partial response, a high SMI index was noted in the tumor bed, confirming a partial pathological response.

On grayscale ultrasound following NAC, response assessment was performed in accordance with RECIST 1.1 criteria [19]. Lesions demonstrating no change in size or morphology compared with pre-treatment imaging, or a size reduction of less than 30%, were classified as no response. A reduction in lesion size of more than 30% with persistence of a solid mass exhibiting a convex contour was categorized as partial response (Figure 3a–d and Figure 4a–c). Cases in which a marked decrease in lesion size was observed but the tumor bed remained clearly identifiable were classified as near-complete/complete response.

### 2.5. MRI Examination

Post-treatment MRI examinations were performed within a comparable time interval to the SMI-US assessments, between completion of NAC and surgery. All patients who underwent post-NAC MRI had also received baseline (pre-NAC) MRI examinations, and treatment response was evaluated through comparative analysis of pre- and post-treatment MRI findings.

Tumor measurements were performed on early and conventional delayed-phase MRI images, acquired at 90 and 360 s following contrast agent administration, respectively. The maximum diameter of the residual enhancing mass or non-mass enhancement was recorded. MRI findings were interpreted in accordance with the BI-RADS criteria. Treatment response on MRI was determined by comparing post-treatment images with baseline studies and categorized as complete/near-complete response, partial response, or no response.

On post-NAC MRI, treatment response was assessed according to RECIST 1.1 criteria [19]. Lesions demonstrating no change in size, morphology, or kinetic features compared with baseline imaging, or a size reduction of less than 30%, were classified as no response. A decrease in lesion size of more than 30% with persistence of a mass exhibiting pathological contrast enhancement was categorized as partial response. Lesions with a marked reduction in size, the absence of early-phase enhancement, and the presence of gradual late-phase non-mass enhancement consistent with a type 1 kinetic pattern suggestive of fibrosis within the tumor bed were classified as near-complete response (Figure 3e–h and Figure 4d,e).

### 2.6. Clinical and Pathology Information

Data including patient age, tumor type, and hormone receptor status; HER-2 and Ki-67 data including patient age, tumor type, hormone receptor status, HER-2 expression status andKi-677 expression levels; and the nuclear grade were meticulously recorded based on the results of the initial mass core biopsy performed at the time of diagnosis. All patients were assessed 1–3 weeks after completing NAC.

The established neoadjuvant chemotherapy regimen at our institution consisted of four initial cycles of doxorubicin in combination with cyclophosphamide followed by twelve cycles of paclitaxel for patients with HER-2 negative and hormone receptor-positive or triple-negative breast cancer (TNBC). Patients with HER-2 positive breast cancer received six cycles of docetaxel and carboplatin, with the possible addition of pertuzumab.

In this study, pCR was defined as the absence of both residual invasive carcinoma and DCIS in the surgical specimen. Although the widely accepted definition of pCR permits the presence of residual DCIS, residual DCIS is often interpreted as a near-complete response on imaging and may affect conventional MRI and grayscale ultrasound assessments, leading to discrepancies between radiologic and pathologic response evaluation. The definition of pCR influences the diagnostic performance of MRI in detecting residual disease; when residual DCIS is included within the definition of pCR, pooled sensitivity tends to increase, whereas pooled specificity decreases [20]. Therefore, to enable a more accurate comparison of SMI with other imaging modalities in predicting true complete response, we adopted a stricter definition of pCR, requiring the absence of both invasive carcinoma and DCIS.

### 2.7. Statistical Analysis of Data

Skewness and kurtosis coefficients were calculated to assess whether continuous variables followed a normal distribution. The calculated skewness and kurtosis values ranged from −1.5 to +1.5. Categorical variables were presented as frequencies (n, %) and continuous variables as means and standard deviations. The normality of data distribution was evaluated using the Kolmogorov–Smirnov test. Comparisons of continuous variables between two groups were performed using the independent sample *t*-test, while comparisons between more than two groups were performed using one-way ANOVA.

Chi-squared tests (Pearson’s chi-squared test and Fisher’s exact test) were used to compare breast-pCR rates based on categorical variables. The relationship between two continuous variables was analyzed using Pearson’s correlation. In univariate analyses, variables associated with breast-pCR were entered into a stepwise multiple logistic regression model, which identified four independent predictors: MRI, US, HER-2, and SMI. The odds ratio (OR) and 95% confidence interval (CI) were reported for each independent variable.

Receiver Operating Characteristic (ROC) analysis was used to evaluate the predictive ability of the independent variables for breast-pCR. The area under the ROC curve (AUC), sensitivity, specificity, positive predictive value (PPV), negative predictive value (NPV), and accuracy were calculated for each variable and model, and the optimal threshold was determined using the Youden index. In addition, the strength of the relationship between dependent and independent variables was quantified using the Nagelkerke R^2^ statistic.

All results were evaluated with a 95% confidence interval, and statistical significance was defined as *p* < 0.05 (two-sided). Statistical analyses were performed using SPSS (Statistical Package for the Social Sciences) version 27 (IBM Corp., Armonk, NY, USA) and R software (version 4.3.1; https://www.r-project.org/).

## 3. Results

### 3.1. Patient, Tumor and Response Characteristics

A total of 115 breast cancer patients with a median age of 51 years (range, 30–75) were included in the study. Among the tumors, 63 (55%) were high-grade and the majority (95%) had invasive ductal carcinoma histology. Analysis of receptor status revealed that 71 patients (62%) were ER-positive, 57 (50%) were PR-positive, and 51 (44%) were HER-2 positive. The median Ki-67 expression level was 40% (range, 5–90%), with 67 patients (58%) demonstrating Ki-67 levels of 40% or higher (Table 1).

Based on the molecular classification of breast cancer, 71 patients (62%) were classified as luminal, while 44 (38%) were classified as non-luminal. Following NAC, a complete pathological response in the breast was achieved in 49 patients (43%). Among the 90 patients with primary axillary involvement, complete axillary response was observed in 48 cases (Table 1).

### 3.2. SMI Index Level

**Figure 5:**
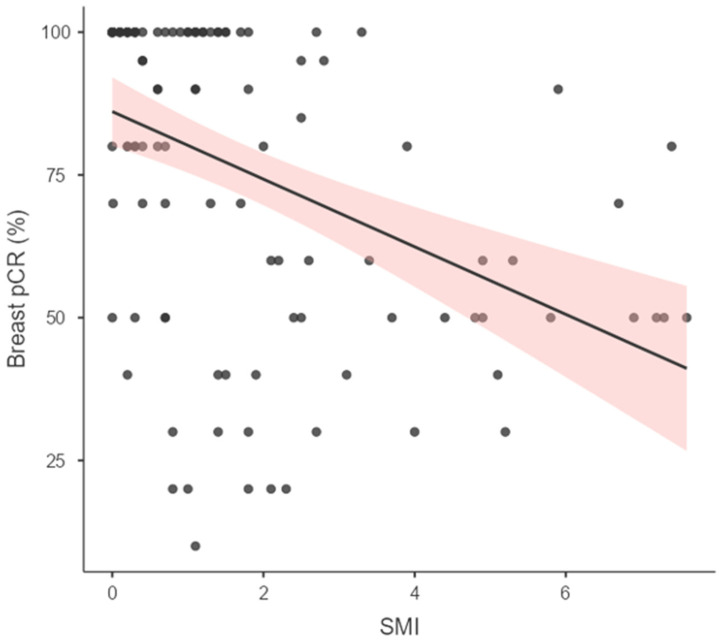
The graph shows the negative correlation between pathological complete response (pCR) and post-neoadjuvant chemotherapy (NAC) Superb Microvascular Imaging (SMI) index measurement. The dots represent the distribution of patients.

The mean SMI value was calculated as 1.70 ± 1.95, with a median of 1.1 (range: 0.0–7.6). Patients who achieved pCR had significantly lower SMI levels. A statistically significant negative correlation was identified between breast-pCR and SMI levels (r = −0.407; *p* < 0.001) (Figure 5).

**Figure 6:**
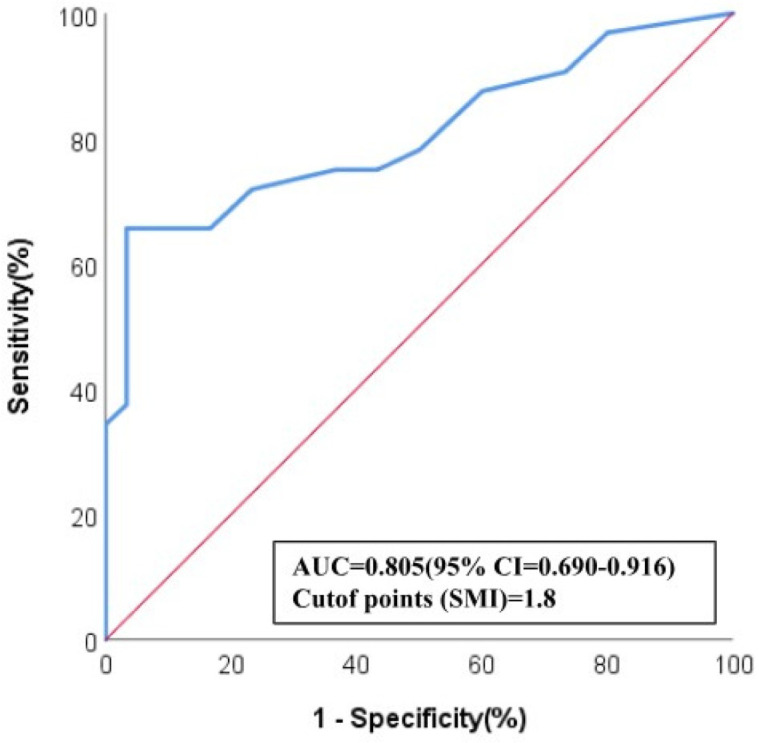
Receiver operating characteristic (ROC) analysis demonstrating the diagnostic performance of Superb Microvascular Imaging (SMI) in predicting pathological complete response (pCR). The blue curve represents the ROC curve of SMI, whereas the red line denotes the line of no discrimination. The area under the curve (AUC) was 0.805 (95% CI: 0.690–0.916), and the optimal cut-off value for SMI was 1.8.

ROC analysis revealed that SMI demonstrated a strong ability to predict breast-pCR, with an AUC of 0.804 (95% CI: 0.726–0.882). The optimal threshold for SMI in predicting pCR was determined to be 1.8 (Figure 6).

When analyzing SMI levels in relation to patient characteristics other than pCR, lower SMI levels were observed in patients aged over 50 years (*p* = 0.031), in patients with negative Progesterone Receptor (PR) status (*p* = 0.019), and in cases demonstrating complete response on US and MRI (*p* < 0.001). However, no statistically significant correlation was found between axillary pCR and tumor SMI values based on the breast tumor bed (*p*> 0.05) (Table 2).

### 3.3. Variables Associated with Breast-pCR Presence

#### 3.3.1. Univariate Analysis Results

Breast hormone receptor (ER and PR) negativity, HER-2 positivity, high Ki-67 expression (≥40%), non-luminal molecular subtype, and radiological response to treatment (assessed by US and MRI) following NAC were significantly associated with the presence of breast-pCR (*p* < 0.001, *p* < 0.01, and *p* < 0.05, respectively) (Table 2).

#### 3.3.2. Diagnostic Performance of Independent Parameters (US, MRI, SMI, and HER-2) in Predicting Breast Pathologic Response

For the SMI index with a threshold of 1.8, sensitivity, specificity, and accuracy were calculated as 93.9%, 55%, and 71.3%, respectively. The sensitivity of SMI in detecting pCR was higher than that of US and MRI in all patients, as well as in the luminal and non-luminal subgroups. However, US demonstrated greater accuracy in identifying pCR presence in the total patient group and in the non-luminal subgroup. In the luminal group, US and MRI accuracy rates were similar and higher than that of SMI. Conversely, in the non-luminal group, SMI accuracy was higher than MRI (Table 3).

### 3.4. Multivariate Analysis Results

Variables found to be significantly associated with breast-pCR in the univariate analyses were included in the stepwise multiple logistic regression model. The final regression model was achieved after four steps (χ^2^ = 60.758, *p* < 0.001). In the final model, the independent predictors of breast-pCR were as follows: decreased SMI levels [OR = 0.475 (95% CI: 0.278–0.812); *p* = 0.007], HER-2positivity [OR = 3.14 (95% CI: 1.103–8.933); *p* = 0.032], complete breast response observed on US [OR = 4.036 (95% CI: 1.298–12.553); *p* = 0.016], and complete breast response observed on MRI [OR = 3.363 (95% CI: 1.029–10.992); *p* = 0.045]. When the Nagelkerke R^2^ statistic was examined, it was determined that the combination of US and SMI explained 47.6% of the variance in the presence of pCR in the breast. This explanatory power increased to 55.1% when HER-2 and MRI were added to the model (Table 4).

### 3.5. ROC Analyses of Multivariate Logistic Regression Models

Three models created using multivariate binary logistic regression analysis were evaluated for their ability to predict breast-pCR (Table 5):

Model 1 (US and SMI): Sensitivity, specificity, and accuracy rates were 71.4%, 84.8%, and 79.1%, respectively.
Model 2 (Model 1 plus HER-2 positivity): Sensitivity, specificity, and accuracy rates were 85.7%, 77.3%, and 80.9%, respectively.
Model 3 (Model 2 plus MRI): Sensitivity, specificity, and accuracy rates were 89.8%, 75.8%, and 81.7%, respectively.

**Figure 7:**
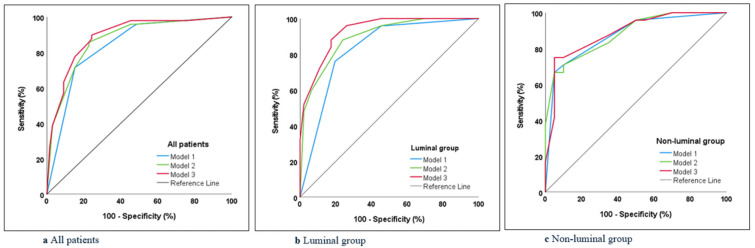
Receiver Operating Characteristic (ROC) curves of three models created to predict pathological complete response (pCR) are shown, and it is seen that the model with the highest accuracy is Model 3 (ultrasound (US) + Superb Microvascular Imaging (SMI) + positive Human Epidermal Growth Receptor 2 (HER-2) status + Magnetic Resonance Imaging (MRI)). (**a**) All patients, (**b**) luminal group, (**c**) non-luminal group.

Based on the ROC analysis, Model 3 demonstrated the highest AUC (AUC = 0.89; 95% CI: 0.829–0.95), making it the most accurate model for distinguishing pCR from non-pCR. Model 3 accurately predicted pCR presence in 89% of cases, with accuracy rates of 84.5% in the luminal group and 84.1% in the non-luminal group (Table 5) (Figure 7).

## 4. Discussion

NAC is a well-established treatment strategy aimed at downstaging breast cancer to enable less invasive surgical approaches [21]. Accurate assessment of treatment response is therefore essential for guiding clinical decision-making. Although multimodal imaging techniques such as US, MG, and MRI are widely used, there remains a need for methods that can more precisely evaluate tumor microvascular changes. Superb Microvascular Imaging (SMI), with its ability to detect low-velocity microvascular flow using advanced filtering techniques, represents a promising tool in this context [10,22].

Previous studies have demonstrated that SMI improves the specificity and diagnostic accuracy of differentiating benign and malignant breast lesions compared to grayscale US [11,16,23,24,25]. Furthermore, the integration of SMI with grayscale imaging has been shown to enhance overall diagnostic performance [13,15]. SMI has also been investigated as a noninvasive imaging biomarker reflecting tumor biology, with higher vascular indices reported in non-luminal breast cancer subtypes [26]. These findings support the concept that SMI-derived vascularity reflects underlying tumor aggressiveness and angiogenic activity.

In the present study, the observed association between lower post-treatment SMI index values and pathological complete response (pCR) suggests that reduced microvascularity following NAC may reflect effective tumor regression. This is consistent with prior evidence indicating that vascular and elasticity parameters correlate with tumor biology and treatment response [27]. The lack of association between SMI and axillary pCR may be explained by the fact that SMI assessment was primarily focused on the primary tumor bed. Additionally, discordant responses between primary tumors and metastatic lymph nodes have been reported and may reflect tumor heterogeneity and differences in the tumor microenvironment.

The higher diagnostic performance of SMI in non-luminal subtypes may be attributed to the increased angiogenic activity typically observed in these tumors. In invasive breast cancer, the vascular index derived from SMI at baseline has also been reported as a prognostic marker, with higher values observed in more aggressive histological types [10,26,28]. These findings suggest that SMI-derived vascular parameters may reflect underlying tumor biology, including angiogenic activity and treatment sensitivity across molecular subtypes. This supports SMI as a functional imaging biomarker of treatment response. Its integration into multimodal imaging may further improve diagnostic accuracy.

Previous studies have shown that elastography and Doppler US performed before or during NAC can serve as prognostic markers for treatment response [4,8]. Kim et al. reported that Doppler US could detect residual tumor in cases where MRI suggested near-complete response, highlighting the limitations of MRI in certain clinical scenarios [6]. Similarly, larger cohort studies have demonstrated that the addition of Doppler US reduces the false-negative rate of MRI [7]. In this context, the incorporation of SMI into post-treatment imaging may provide complementary information and enhance the overall predictive accuracy of imaging-based response assessment.

Contrast-enhanced ultrasound (CEUS) studies have also demonstrated significant associations between vascular parameters and treatment response during and after NAC [18,29,30].

SMI enables superior visualization of low-flow microvascular structures compared to color/power Doppler US (CDUS/PDUS) due to advanced motion artifact suppression [10,22]. While conventional Doppler techniques are limited in detecting very small vessels and slow flow [7,10,22], SMI improves the detection of early-stage malignant lesions, particularly those <1 cm, and enhances diagnostic accuracy when combined with grayscale US [9,31,32].

SMI-derived vascular indices correlate with histopathological markers of angiogenesis [17], and increased vascularity assessed by Doppler techniques has been associated with Progesterone Receptor negativity in breast cancer [33].

Unlike CEUS, SMI does not require contrast administration and can still provide detailed information on tumor microvascularity [34,35]. This makes SMI a practical and noninvasive alternative, particularly in patients where contrast use is limited or contraindicated.

MRI assessment after NAC may be affected by treatment-related changes such as fibrosis and inflammation, which can lead to persistent contrast enhancement and false-positive interpretations [36,37]. Additionally, anti-angiogenic therapies may alter vascular permeability and result in underestimation of treatment response on MRI [38,39]. Given its ability to directly visualize microvascular flow without reliance on contrast kinetics, SMI may help overcome some of these limitations. However, the performance of SMI in the setting of anti-angiogenic therapy remains insufficiently studied and warrants further investigation.

From a technical perspective, the use of a lower velocity scale in this study aimed to improve the detection of low-flow microvascular signals within the post-treatment tumor bed. While higher velocity scales have been commonly used in previous studies [4,25,28], these findings suggest that optimization of SMI parameters according to the treatment stage is critical and highlight the need for standardized imaging protocols in future studies.

This study has several limitations. Its retrospective single-center design and relatively small sample size may limit the generalizability of the findings. The exclusion of cases in which the tumor bed could not be visualized after NAC may introduce selection bias. Additionally, SMI assessment was limited to two-dimensional imaging and may be affected by technical artifacts, particularly in large or deeply located lesions. Despite these limitations, this study contributes to the limited body of literature on post-treatment SMI assessment and supports its potential role as part of a multimodal imaging strategy for evaluating treatment response after NAC.

The radiological prediction of pCR in breast cancer, potentially enabling non-operative surveillance after NAC, is expected to become increasingly important in future clinical practice [40]. In this context, accurate and reproducible assessment of tumor vascularity is critical. Prospective techniques that allow evaluation of vascularity across the entire tumor volume in three dimensions may further improve the effectiveness of NAC response assessment. In addition, recent advances in AI-based segmentation, including methods designed to be robust against annotation errors, may further enhance the reliability and standardization of ultrasound-based quantitative analyses such as SMI, particularly through automated tumor bed delineation, representing a promising direction for future radiomics studies [41].

## 5. Conclusions

This study showed low SMI scores, complete response findings on grayscale US, complete response on MRI, and HER-2 positivity, which emerged as powerful independent predictors of pCR following NAC. SMI demonstrates exceptional accuracy in predicting treatment response, particularly in the HER-2 positive or triple-negative breast cancers. Notably, SMI, particularly when combined with grayscale US, may serve as a noninvasive and quantitative imaging biomarker to predict pCR after NAC for invasive breast cancers.

## Figures and Tables

**Table 1:** Baseline clinicopathologic characteristics of the study population.

Variables	n (%)
**All**	115 (100)
**Age (years), group**	
≤50	57 (49.6)
>50	58 (50.4)
**Tumor type**	
IDC	109 (94.8)
Other	6 (5.2)
**Grade**	
I/II	52 (45.2)
III	63 (54.8)
**ER**	
Positive	71 (61.7)
Negative	44 (38.3)
**PR**	
Positive	57 (49.6)
Negative	58 (50.4)
**HER-2**	
Positive	51 (44.3)
Negative	64 (55.7)
**Ki-67 expression**	
Positive (≥40%)	67 (58.3)
Negative (<40%)	48 (41.7)
**Molecular subgroup**	
Luminal HER-2(-)	41 (35.7)
Luminal HER-2(+)	30 (26.1)
HER-2(+)	21 (18.3)
Triple negative	23 (20)
**Molecular subgroup**	
Luminal	71 (61.7)
Non-luminal	44 (38.3)
**Breast-pCR**	
Yes	49 (42.6)
No	66 (57.4)
**Axilla-pCR (n = 90)**	
Yes	48 (53.3)
No	42 (46.7)

Categorical variables are presented as number (n) and percentage (%). **IDC**, invasive ductal carcinoma; **ER**, Estrogen Receptor; **PR**, Progesterone Receptor; **HER-2**, Human Epidermal Growth Receptor 2; **pCR**, pathological complete response.

**Table 2:** SMI levels and breast-pCR rates based on patient clinicopathologic characteristics. Post-NAC SMI index levels differed significantly according to age and Progesterone Receptor (PR) status, with higher values observed in patients aged ≤50 years and in PR-positive tumors. Breast-pCR rates were significantly associated with tumor grade, ER, PR, HER-2 status, Ki-67 expression, and molecular subtype. Notably, lower SMI index levels were significantly associated with breast-pCR (*p* < 0.001). In contrast, SMI index levels were not significantly associated with axillary pCR (*p* = 0.354). Additionally, SMI levels were significantly lower in patients classified as pCR on ultrasound and MRI evaluations (*p* < 0.001).

			SMI		Breast-pCR	
Variables	Category	n	Mean ± SD	*p*-Value	n (%)	*p*-Value
**All**	**-**	115	1.70 ± 1.95		49 (42.6)	
**Age (years), group**	≤50	57	2.09 ± 2.25	0.031 ^a,^*	28 (49.1)	0.161 ^c^
	>50	58	1.31 ± 1.53		21 (36.2)	
**Tumor type**	IDC	109	1.63 ± 1.92	0.138 ^a^	48 (44)	0.238 ^d^
	Other	6	2.85 ± 2.33		1 (16.7)	
**Grade**	I/II	52	1.94 ± 2.17	0.223 ^a^	16 (30.8)	0.020 ^c,^*
	III	63	1.50 ± 1.75		33 (52.4)	
**ER**	Positive	71	1.94 ± 2.16	0.097 ^a^	25 (35.2)	0.042 ^c,^*
	Negative	44	1.31 ± 1.51		24 (54.5)	
**PR**	Positive	57	2.12 ± 2.17	0.019 ^a,^*	17 (29.8)	0.006 ^c,^*
	Negative	58	1.28 ± 1.62		32 (55.2)	
**HER-2**	Positive	51	1.78 ± 2.03	0.672 ^a^	27 (52.9)	0.045 ^c,^*
	Negative	64	1.63 ± 1.90		22 (34.4)	
**Ki-67 expression**	Positive (≥40%)	67	1.47 ± 1.79	0.136 ^a^	34 (50.7)	0.037 ^c,^*
	Negative(<40%)	48	2.02 ± 2.14		15 (31.3)	
**Molecular subgroup**	Luminal HER-2(-)	41	1.91 ± 2.09	0.366 ^b^	9 (22)	0.011 ^c,^*
	Luminal HER-2(+)	30	1.97 ± 2.28		16 (53.3)	
	HER-2(+)	21	1.51 ± 1.63		11 (52.4)	
	Triple negative	23	1.13 ± 1.40		13 (56.5)	
**Molecular subgroup**	Luminal	71	1.94 ± 2.16	0.097 ^a^	25 (35.2)	0.042 ^c,^*
	Non-Luminal	44	1.31 ± 1.51		24 (54.5)	
**US**	pCR	48	0.94 ± 1.22	<0.001 ^a,^*	36 (75)	<0.001 ^c,^*
	non-pCR	67	1.95 ± 1.84		13 (19.4)	
**MRI**	pCR	34	0.71 ± 0.85	<0.001 ^b,^*	27 (79.4)	<0.001 ^c,^*
	Non-pCR	50	2.27 ± 2.06		9 (18)	
	Absent MRI	31	1.85 ± 2.24		13 (41.9)	
**Breast-pCR**	Yes	49	0.64 ± 0.74	<0.001 ^a,^*		
	No	66	2.49 ± 2.19			
**Axilla-pCR (n = 90)**	Yes	48	1.65 ± 2.01	0.354 ^a^		
	No	42	2.04 ± 2.00			

* *p* < 0.001; **^a^**, Independent sample *t*-test; **^b^**, one-way ANOVA; **^c^**, Pearson’s chi-square test; **^d^**, Fisher’s exact test; *p* values in bold indicate statistical significance (*p* < 0.05). **SD**, standard deviation. Continuous variables are presented as mean ± standard deviation (SD), and categorical variables as number (n) and percentage (%). **IDC**, invasive ductal carcinoma; **ER**, Estrogen Receptor; **PR**, Progesterone Receptor; **HER-2**, Human Epidermal Growth Receptor 2; **US**, ultrasound; **MRI**, Magnetic Resonance Imaging; **pCR**, pathological complete response.

**Table 3:** Diagnostic performance of independent parameters (US, MRI, SMI, and HER-2) in predicting breast-pCR in the overall cohort and according to molecular subtype. For SMI, the optimal cut-off value was determined as <1.8. In the overall cohort, SMI demonstrated the highest sensitivity (93.9%) and negative predictive value (92.3%) for predicting breast-pCR, whereas MRI showed the highest specificity (89.4%) and positive predictive value (79.4%). Ultrasound exhibited balanced sensitivity (73.5%) and specificity (81.8%). HER-2positivity alone showed limited diagnostic performance. In the luminal subgroup, SMI maintained very high sensitivity (96%) but relatively low specificity (54.3%), while MRI provided the highest specificity (89.1%). In the non-luminal subgroup, SMI again demonstrated high sensitivity (91.7%), whereas MRI and US yielded higher specificity (90%). Overall, SMI was characterized by high sensitivity across subgroups, while MRI provided superior specificity for predicting breast-pCR.

Group	Variables	Cut-Off	SE (95% CI)	SP (95% CI)	PPV (95% CI)	NPV (95% CI)	ACC (95% CI)
All	US	pCR	**73.5** (61.1–85.8)	81.8 (72.5–91.1)	75 (62.8–87.3)	80.6 (71.1–90.1)	78.3 (70.7–85.8)
(*n* = 115)	MRI	pCR	55.1 (41.2–69)	**89.4** (82–96.8)	**79.4** (65.8–93)	72.8 (63.2–82.5)	74.8 (66.8–82.7)
	SMI	<1.8	**93.9** (87.2–100)	54.5 (42.5–66.6)	60.5 (49.5–71.5)	**92.3** (83.9–100)	71.3 (63–79.6)
	HER-2	positive	55.1 (41.2–69)	63.6 (52–75.2)	52.9 (39.2–66.6)	65.6 (54–77.3)	60 (51–69)
Luminal	US	pCR	76 (59.3–92.7)	78.3 (66.3–90.2)	65.5 (48.2–82.8)	85.7 (75.1–96.3)	77.5 (67.7–87.2)
(*n* = 71)	MRI	pCR	60 (40.8–79.2)	**89.1** (80.1–98.1)	**75** (56–94)	80.4 (69.5–91.3)	78.9 (69.4–88.4)
	SMI	<1.8	**96** (88.3–104)	54.3 (40–68.7)	53.3 (38.8–67.9)	**96.2** (88.8–100)	69 (58.3–79.8)
	HER-2	positive	64 (45.2–82.8)	69.6 (56.3–82.9)	53.3 (35.5–71.2)	78 (65.4–90.7)	67.6 (56.7–78.5)
Non-luminal	US	pCR	70.8 (52.6–89)	**90** (76.9–100)	**89.5** (75.7–100)	72 (54.4–89.6)	79.5 (67.6–91.5)
	MRI	pCR	50 (30–70)	**90** (76.9–100)	85.7 (67.4–100)	60 (42.5–77.5)	68.2 (54.4–81.9)
(*n* = 44)	SMI	<1.8	**91.7** (80.6–100)	55 (33.2–76.8)	71 (55–86.9)	**84.6** (65–104)	75 (62.2–87.8)
	HER-2	positive	45.8 (25.9–65.8)	50 (28.1–71.9)	52.4 (31–73.7)	43.5 (23.2–63.7)	47.7 (33–62.5)

**SE**, sensitivity; **SP**, specificity; **NPV**, negative predictive value; **PPV**, positive predictive value; **ACC**, accuracy; **CI**, confidence interval; **US**, ultrasound; **MRI**, Magnetic Resonance Imaging; **SMI**, Superb Microvascular Imaging; **HER-2**, Human Epidermal Growth Receptor 2. Bold values indicate the best diagnostic performance (highest sensitivity, specificity, PPV, NPV, or accuracy) for each parameter within the corresponding group.

**Table 4:** Stepwise multiple logistic regression analysis shows an association between SMI index levels and breast-pCR according to clinicopathologic and imaging characteristics. Ultrasound-based pCR assessment emerged as a strong independent predictor of breast-pCR in Step 1 (OR = 12.462, *p* < 0.001). With the addition of SMI in Step 2, both US (OR = 6.124, *p* < 0.001) and SMI (OR = 0.494, *p* = 0.005) remained significant predictors, and model performance improved (R^2^N = 0.476). In Step 3, HER-2positivity was identified as an additional independent predictor (OR = 3.544, *p* = 0.016), further increasing the explanatory power of the model (R^2^N = 0.523). In the final model (Step 4), MRI-based pCR assessment also emerged as an independent predictor (OR = 3.363, *p* = 0.045), alongside US, HER-2 positivity, and SMI. Notably, lower SMI values were independently associated with breast-pCR across all multivariable models (final model OR = 0.475, *p* = 0.007). The final model demonstrated the highest predictive performance (R^2^N = 0.551; χ^2^ = 60.758), indicating that the combined use of imaging parameters and HER-2 status significantly improved the prediction of breast-pCR.

Model	Variables	B	SE	OR (95% CI)	*p*-Value	R^2^_N_	χ2
Step 1	US (pCR)	2.523	0.454	12.462 (5.113–30.369)	<0.001	0.369	36.988
Step 2	US (pCR)	1.812	0.498	6.124 (2.307–16.251)	<0.001	**0.476**	50.347
	SMI	−0.706	0.250	0.494 (0.303–0.806)	0.005		
Step 3	US (pCR)	1.866	0.528	6.465 (2.295–18.213)	<0.001	**0.523**	56.669
	HER-2 (+)	1.265	0.523	3.544 (1.271–9.881)	0.016		
	SMI	−0.772	0.267	0.462 (0.274–0.780)	0.004		
Step 4	MRI (pCR)	1.213	0.604	3.363 (1.029–10.992)	0.045	**0.551**	60.758
	US (pCR)	1.395	0.579	4.036 (1.298–12.553)	0.016		
	HER-2 (+)	1.144	0.534	3.140 (1.103–8.933)	0.032		
	SMI	−0.745	0.274	0.475 (0.278–0.812)	0.007		

**Dependent variables:** Breast-pCR (1, yes; 0, no); **OR**, odds ratio; **SE**, standard error. **US**, ultrasound; **MRI**, Magnetic Resonance Imaging; **SMI**, Superb Microvascular Imaging; **HER-2**, Human Epidermal Growth Receptor 2; **pCR**, pathological complete response. Bold values indicate significant predictors of breast-pCR (*p* < 0.05).

**Table 5:** ROC analysis of multivariate binary logistic regression models for predicting breast-pCR after NAC in the overall cohort and according to molecular subtype (luminal and non-luminal). Model 1 includes US and SMI findings; Model 2 includes HER-2 positivity in addition to Model 1 variables; Model 3 includes MRI in addition to Model 2 variables. Discriminative performance improved progressively with the addition of molecular and imaging parameters. In the overall cohort, the AUC increased from 0.838 in Model 1 to 0.872 in Model 2 and reached 0.890 in Model 3, with a corresponding increase in sensitivity (from 71.4% to 89.8%) and negative predictive value (from 80% to 90.9%). In the luminal subgroup, model performance improved markedly, with the AUC rising from 0.831 in Model 1 to 0.929 in Model 3. Model 3 demonstrated the highest diagnostic accuracy (84.5%) and maintained high sensitivity (88%) and specificity (82.6%). In the non-luminal subgroup, all models showed high discriminative ability (AUC range: 0.874–0.888) with consistently high specificity (95%). Although the incremental improvement between models was less pronounced compared to the luminal group, Model 3 achieved higher sensitivity (75%) and overall accuracy (84.1%). Model 3 demonstrated the highest overall diagnostic accuracy.

Variables	AUC (95% CI)	SE (95% CI)	SP (95% CI)	PPV (95% CI)	NPV (95% CI)	ACC (95% CI)
**All**						
Model 1	**0.838** (0.764–0.912)	71.4 (58.8–84.1)	84.8 (76.2–93.5)	77.8 (65.6–89.9)	80 (70.6–89.4)	79.1 (71.7–86.6)
Model 2	**0.872** (0.806–0.937)	85.7 (75.9–95.5)	77.3 (67.2–87.4)	73.7 (62.3–85.1)	87.9 (79.5–96.3)	80.9 (73.7–88.1)
Model 3	**0.890** (0.829–0.950)	89.8 (81.3–98.3)	75.8 (65.4–86.1)	73.3 (62.1–84.5)	90.9 (83.3–98.5)	81.7 (74.7–88.8)
**Luminal**						
Model 1	**0.831** (0.734–0.928)	76 (59.3–92.7)	80.4 (69–91.9)	67.9 (50.6–85.2)	86 (75.7–96.4)	78.9 (69.4–88.4)
Model 2	**0.896** (0.822–0.970)	88 (75.3–100)	76.1 (63.8–88.4)	66.7 (50.6–82.8)	92.1 (83.5–100)	80.3 (71–89.5)
Model 3	**0.929** (0.874–0.985)	88 (75.3–100)	82.6 (71.7–93.6)	73.3 (57.5–89.2)	92.7 (84.7–100)	84.5 (76.1–92.9)
**Non-luminal**						
Model 1	**0.874** (0.769–0.979)	66.7 (47.8–85.5)	95 (85.4–100)	94.1 (82.9–100)	70.4 (53.1–87.6)	79.5 (67.6–91.5)
Model 2	**0.882** (0.787–0.978)	66.7 (47.8–85.5)	95 (85.4–100)	94.1 (82.9–100)	70.4 (53.1–87.6)	79.5 (67.6–91.5)
Model 3	**0.888** (0.789–0.986)	75 (57.7–92.3)	95 (85.4–100)	94.7 (84.7–100)	76 (59.3–92.7)	84.1 (73.3–94.9)

**AUC**, area under curve; **SE**, sensitivity; **SP**, specificity; **NPV**, negative predictive value; **PPV**, positive predictive value; **ACC**, accuracy; **CI**, confidence interval. **Model 1**, US and SMI are included; **Model 2**, HER-2 positivity added to Model 1; **Model 3**, MRI added to Model 2.

## Data Availability

The datasets generated and/or analysed during the current study are not publicly available due to ethical considerations/institutional policies but are available from the corresponding author on reasonable request.

## Abbreviations

CDUS	Color Doppler Ultrasound
CEUS	Contrast-Enhanced Ultrasound
DCIS	Ductal Carcinoma In Situ
DR	Dynamic Range
ER	Estrogen Receptor
FPS	Frames Per Second
HER-2	Human Epidermal Growth Receptor 2
IDC	Invasive Ductal Carcinoma
MG	Mammography
MRI	Magnetic Resonance Imaging
NAC	Neoadjuvant Chemotherapy
pCR	Pathological Complete Response
PDUS	Power Doppler Ultrasound
PET	Positron Emission Tomography
PR	Progesterone Receptor
ROC	Receiver Operating Characteristic
ROI	Regions of Interest
SE	Sensitivity
SMI	Superb Microvascular Imaging
SP	Specificity
TNBC	Triple-Negative Breast Cancer
US	Ultrasound
